# How Green Space Justice in urban built-up areas affects public mental health: a moderated chain mediation model

**DOI:** 10.3389/fpubh.2024.1442182

**Published:** 2024-10-02

**Authors:** Tian Dong, Qikang Zhong, Bangguo Yue

**Affiliations:** School of Architecture and Art, Central South University, Changsha, China

**Keywords:** Green/Blue Space Justice, physical and psychological responses, mental health, structural equation modeling, moderation effect, healthy city construction

## Abstract

**Background:**

Green and blue spaces, as crucial components of urban ecosystems, significantly impact the physical and mental health of residents. However, the mechanisms through which Green/Blue Space Justice influence residents’ health remain unclear.

**Methods:**

This study aims to explore the impact of green spaces on public psychological responses, physical activity, and mental health from a justice perspective, and to examine the moderating role of blue spaces in this relationship. The research was conducted in selected communities within the Chang-Zhu-Tan urban agglomeration in Hunan Province, China. A total of 801 valid questionnaires were collected through field visits and online surveys. The study uses an improved Gaussian-based two-step floating catchment area (2SFCA) method to assess green space accessibility. Data were analyzed using structural equation modeling (SEM) and moderation effect analysis to reveal the relationships between variables.

**Results:**

The findings indicate that Green Space Justice has a significant positive impact on psychological responses, physical activity, and mental health; psychological responses and physical activity play crucial mediating roles between Green Space Justice and mental health; and Green Space Justice significantly affects mental health through a chain mediation path involving psychological responses and physical activity. Moreover, Blue Space Justice significantly moderates the impact of Green Space Justice on psychological responses and physical activity, but does not have a significant direct impact on mental health.

**Conclusion:**

This study enriches the theory of Green Space Justice by revealing the mechanisms through which it influences mental health via psychological responses and physical activity. It provides a scientific basis for the development of healthy cities. Additionally, it recommends that urban planning should prioritize the equitable distribution and high accessibility of both green and blue spaces to comprehensively enhance residents’ physical and mental well-being. Policymakers should consider prioritizing the accessibility of high-quality green spaces for vulnerable communities during urban renewal and expansion processes to reduce social health inequalities and promote broader public health outcomes.

## 1 Introduction

Rapid urbanization and fast-paced, unhealthy lifestyles are threatening human physical and mental health and quality of life (1). Globally, over one billion people are affected by mental health issues (2). The COVID-19 pandemic in recent years has exacerbated mental health problems worldwide, including anxiety, confusion, and stress (3). A study from Mexico confirmed that the rates of suicide and depression have been rising in recent years (4). In addition, the widespread use of the internet has impacted the mental health of adolescents, who are in a critical stage of physical and psychological development and are particularly susceptible to external influences. A study from China indicates that stress, anxiety, and depression among adolescents have become significant social issues in recent years (5). Although the factors leading to the deterioration of human physical and mental health are complex and multifaceted, increasing evidence suggests that urban green and blue spaces have a positive impact on physical and mental health. More accessible green spaces help reduce residents’ stress and psychological distress (6–9). Nguyen et al. (10) systematically summarized the effects of Nature prescriptions on mental health and physical activity, highlighting the promising role of green and blue spaces in treating mental health issues.

Concurrently, urban green and blue spaces have become a focal point in environmental justice research (11). Environmental justice, particularly Green Space Justice, refers to the equitable distribution of green spaces among different socio-economic groups. It emphasizes the need to ensure that low-income, minority, and other socially disadvantaged groups have fair access to public amenities such as parks and green spaces (12). However, the advancement of urbanization has led to a reduction in the availability and equitable distribution of urban green spaces (13, 14), potentially exacerbating health inequalities (15). While increasing green space exposure can improve residents’ health and well-being, the green space premium effect in urban areas creates disparities in the health benefits enjoyed by residents of different socio-economic statuses, thereby deepening health inequalities in megacities. Numerous studies support this point: Matthias et al. used sensing, OpenStreetMap, and census data to reveal significant disparities in the distribution of green spaces among populations in German cities, with notable urban–rural differences and significant influence from household economic status (16, 17). Mikel’s study involving 1,738 children aged 6 to 8 and 1,449 children aged 10 to 12 demonstrated that economic income and educational differences lead to inequitable exposure to green and blue spaces (18), resulting in vulnerable groups being more exposed to harmful environments (19). This hinders the achievement of human physical and mental health and well-being, as well as the long-term stable development of society. Although numerous studies have demonstrated the positive effects of green spaces on mental health, research on the equitable distribution of green spaces remains limited. These studies often focus solely on the quantity or accessibility of green spaces relative to residential areas, while neglecting other important factors such as the size and quality of the green spaces (20). Based this background, the present study first examines the direct impact of green spaces on public psychological responses, physical activity, and mental health. It then analyzes the distribution of these green space resources from a justice perspective and evaluates the moderating role of blue space equity in this process. This research is of significant importance for promoting human well-being, planning healthy cities, and fostering harmonious societies.

### 1.1 The relationship between green spaces and residents’ mental health

Urban green spaces encompass a wide variety of areas, such as parks, community gardens, cemeteries, rooftop gardens, vertical gardens, lawns, street trees, and small engineered green facilities (21). These spaces play a crucial role in improving residents’ mental health (22, 23), with an increase in *per capita* green space significantly reducing levels of depression (24). Increasingly, research is focusing on the relationship between green spaces and mental health.

Das et al. (25) conducted a year-long study involving bi-weekly surveys of 8,253 adults from 50 U.S. states and found that green spaces can improve negative emotions and reduce the occurrence of mental illnesses. Spano et al. (26) investigated the relationship between green exposure and mental health among 3,886 Italian respondents during the COVID-19 lockdown, discovering that green spaces positively affect anxiety, fear, irritability, and sleep disorders. de la Osa et al. (27) conducted a 3–11 year longitudinal study on 539 community children in Barcelona, analyzing the relationship between green exposure at schools and residences and psychological anxiety. They concluded that increased exposure to green spaces, especially at school, is crucial for children’s mental health (27). Liu et al. (28) evaluated the relationship between residential green exposure and depression using a multilevel linear model with data from 15,826 families across 401 communities in 158 Chinese cities, encompassing 21,086 sample members. They found that greater green space exposure helps reduce the incidence of depression and other mental illnesses (28). Although many studies have demonstrated the positive effects of green spaces on mental health, no research has yet combined indicators such as park quality, park availability, and greening coverage. This gap limits the ability to provide information for equitable urban green space planning, making it difficult for urban planners and policymakers to identify and address issues of environmental justice and health equity in cities (29). Therefore, this study holds significant practical relevance.

### 1.2 The mediating role of physical activity and psychological responses in green spaces

Green spaces can provide urban residents with areas for social interaction, encouraging various forms of communication and engagement (30), and promoting a range of physical activities (31, 32). Higher exposure to community green spaces is associated with higher prevalence rates of physical activity among residents (33). Numerous studies have discussed the impact of physical activity in green spaces on mental health, yielding predominantly positive results. For example, walking or simply spending time in green spaces can immediately improve mood and state anxiety (34), while physical activity can enhance residents’ self-esteem (35) and positively influence mental health (36). Moreover, Kajosaari and Pasanen (37) employed a spatial method using Public Participation Geographic Information Systems (PPGIS) to explore the effects of different outdoor sports environments on mental health, finding that physical activities conducted in green spaces are more effective in alleviating stress and promoting relaxation. Naghibi et al. (38) utilized the EMOTIV system and visual questionnaires to capture individuals’ perceptions and observations of green space landscapes. They employed two psychological scales to assess attention and stress levels, finding that increased exposure to green spaces made respondents feel more comfortable, relaxed, and cheerful. This emphasizes the role of green spaces in restoring mental health and enhancing mood (38). Similarly, a study by Grellier et al. (39) found that among 22 million residents in England aged 16 and over, at least one weekly green space activity effectively prevented 10,552 cases of major depression. Therefore, this study posits that physical activity in green spaces serves as a mediating factor between green space exposure and residents’ mental health.

Additionally, psychological responses can significantly impact mental health. For instance, Zheng et al. (40) conducted a study on the mental health of 3,501 older adult individuals in 146 Chinese cities and found that psychological responses significantly influence mental health. Kim et al. (41) analyzed 661 adolescents under 19 years old in Korea and found that leisure perception, relaxation, and green space public leisure areas significantly impact adolescents’ well-being. This may be due to the fact that psychological responses can influence mental health by stimulating hormone secretion. Positive psychological responses can induce the release of dopamine, oxytocin, serotonin, and endorphins (42), all of which have beneficial effects on mental health (43, 44). Vegaraju and Amiri (45) analyzed the 2011–2019 BRFSS dataset to study older adults in the United States aged 65 and above, demonstrating a positive correlation between green spaces and the subjective well-being of urban older adult residents, as well as a reduction in psychological distress. Similarly, Delgado-Serrano et al. conducted a study involving 632 individuals in Spain, further confirming that green spaces generally enhance well-being, support mental health, and reduce psychological distress. However, the benefits of green spaces are not consistent across different urban environments (46). Therefore, this study hypothesizes that psychological responses in green spaces are another mediating factor between Green Space Justice and residents’ mental health.

### 1.3 The chain mediating role of green space physical activity and green space psychological activity

Previous research has indicated that green space physical activity and green space psychological responses might serve as independent mediators of the effect of green spaces on residents’ mental health. However, other studies suggest the existence of a more complex mediation mechanism between these factors. Research has shown that the frequency of exercise is significantly related to self-esteem, stress, depression, and happiness; frequent participation in exercise can make individuals feel more pleasant and relaxed (47–49), while a reduction in exercise frequency can decrease subjective well-being (50). Harada et al. (51) conducted an in-depth analysis of the effects of exercise duration, frequency, and intensity on psychological responses, demonstrating that physical activity is crucial for improving the daily life satisfaction of older adults. The frequency of exercising for more than 30 min significantly impacts subjective health and happiness in older adults (52), with the relationship between physical activity and psychological responses being more pronounced for males, older adults, unmarried individuals, rural residents, those lacking social security, those with higher levels of depression, and those of lower socioeconomic status (53). When individuals experience negative psychological activities such as guilt or shame, they are more likely to spend time on outdoor activities to alleviate anxiety. Conversely, when people are in a peaceful state of mind, they may engage in green space activities to seek relaxation and other positive psychological benefits (54). These actions have been shown to be beneficial to mental health. Therefore, this study hypothesizes that green space physical activity and green space psychological responses play a chain mediating role in the impact of green spaces on residents’ mental health.

### 1.4 The synergistic moderating role of blue spaces

Different forms of water bodies, such as rivers, ponds, and lakes, are referred to as blue spaces (55). In recent years, many scholars have explored the relationship between blue spaces and human physical and mental health, social relationships, and cultural perceptions (56). Proximity to blue spaces has a positive impact on human and social ecological health, with larger peripheral blue spaces being more beneficial (57). White et al. (58) proposed a model systematically explaining how proximity to blue spaces can enhance health and well-being. Additionally, a review of extensive literature has found a positive correlation between outdoor blue spaces and the health of older adults (59). A study from Greece, analyzing vast data, demonstrated that exposure to blue spaces significantly reduces natural mortality rates and deaths from cardiovascular, respiratory, neurological, cerebrovascular, and ischemic heart diseases (60). Although the area of oceans far exceeds that of inland freshwater bodies, research shows that more than 50% of the global population lives within 3 km of freshwater. Clearly, studying the relationship between freshwater blue spaces and the health and well-being of urban residents holds greater significance than research on coastal blue spaces (61). However, many studies to date have focused on clarifying the relationship between coastal blue spaces and health, with much less attention given to freshwater blue spaces within urban areas (62). As a result, there is currently little knowledge about the potential of different types and quantities of freshwater blue spaces to promote health and well-being (63, 64). Therefore, incorporating blue spaces into research is of significant practical importance.

Increasingly, research on blue spaces has focused on mental health. Chen and Yuan (65) studied older adult residents in Guangzhou and found that exposure to blue spaces positively affects mental health by improving mood, alleviating depression and anxiety, and aiding in the recovery from psychological disorders. Moreover, blue space availability was found to be more effective than green space availability in reducing depression rates among the older adult (66).

Research has also begun to focus on the relationship between physical activity, mental health, and blue spaces. Murrin et al. (67) studied 350 participants and found that physical activity could explain the association between inland blue spaces and mental health outcomes. Studies have shown that even 3 min of visual contact with blue spaces can promote mental health (68), and swimming in open water can improve mood, mental health, and alleviate the distress caused by mental illnesses (69). Although many studies have focused on specific age groups (children/adults/older adult), there is evidence that blue spaces benefit the physical activity and health and well-being of all age groups (70).

Beyond studying the independent moderating effects of blue spaces, Elliott et al. (71) analyzed data from 15,917 cross-sectional studies in 18 countries, finding that the connection between nature and residents’ health is primarily achieved through exposure to green and blue spaces, providing a theoretical basis for promoting health and preventing diseases through these spaces. Marques et al. (72) studied 1,359 mother-newborn pairs and found that exposure to green and blue spaces during pregnancy is related to infant health, and exposure during infancy can affect mental health not only at present but also into adulthood (73). Vegaraju and Amiri (45) used Behavioral Risk Factor Surveillance System (BRFSS) data from phone interviews to demonstrate that exposure to green and blue spaces positively affects the physical and mental health of the older adult in Washington, effectively reducing their psychological distress.

Similar to green spaces, blue spaces also play a moderating role in physical activity and mental health. Therefore, studying the synergistic effects of blue spaces alongside green spaces on physical activity and mental health is of significant importance.

### 1.5 The current study

In reviewing the existing literature, this study found that although there is an increasing focus on mental health, research on physical health remains more prevalent, and the evidence on mental health is still insufficient. Moreover, while some scholars have analyzed green and blue spaces together, many prefer to study either green or blue spaces independently, with few studies examining their combined effects. At the same time, existing evidence indicates that urban green and blue spaces are beneficial to mental health; however, the optimal quantity, location, duration, type, and quality of these spaces remain unknown (74). Therefore, it is necessary to conduct an in-depth analysis of the attributes of green and blue spaces, including their quantity, area, and accessibility.

Given this research context, this study uses urban remote sensing data and questionnaire interview data to explore the mechanisms by which Green Space Justice affects mental health. By employing structural equation modeling and moderation models, this research examines the mediating roles of green space physical activity and green space psychological responses, along with the moderating effect of Blue Space Justice. This study explores the mechanisms through which green space influences mental health from a justice perspective, addressing gaps in the existing research. This study aims to answer the following key questions: how does Green Space Justice affect residents’ mental health through the mediating mechanisms of psychological responses and physical activity in urban built-up areas? Additionally, does Blue Space Justice play a moderating role in this pathway? Specifically, this study will examine: (1) the direct impact of Green Space Justice on residents’ psychological responses, physical activity, and mental health; (2) the mediating role of psychological responses and physical activity between Green Space Justice and mental health; and (3) the moderating role of Blue Space Justice in the relationship between Green Space Justice and psychological responses, physical activity, and mental health.

## 2 Materials and methods

### 2.1 Study area

**Figure 1:**
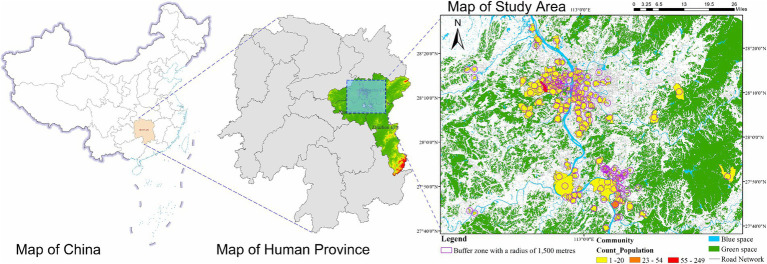
Study area.

This study employs a random visit approach, selecting random communities within the built-up areas of the Changsha-Zhuzhou-Xiangtan City Group (CZXCG) in Hunan Province as the research area (Figure 1). This region is located in south-central China, offering a favorable geographical position and convenient transportation, making it a significant economic and cultural area in the country.

The CZXCG has numerous advantages and holds significant importance. Firstly, as an important urban cluster in south-central China, the economic development of CZXCG has been rapid. Changsha, as the provincial capital, has demonstrated strong economic growth and technological innovation capabilities, driving the development of the entire urban cluster. Secondly, despite being a highly urbanized area, the CZXCG retains a rich natural landscape, including several large parks, green spaces, and water bodies, providing residents with a favorable ecological environment. This blend of natural environments and high urbanization makes the CZXCG an ideal area for studying Green Space Justice and its impact on public physical activity and mental health.

Additionally, the CZXCG is densely populated, with a high level of urbanization and diverse community types. It includes highly developed central urban areas as well as relatively new surrounding communities, offering a diverse sample for research. This diversity helps to deeply explore the differences in green space distribution and utilization across different community types. More importantly, as urbanization accelerates, the CZXCG faces numerous challenges and opportunities in green space planning and construction. Studying the impact of Green Space Justice on residents’ physical and mental health in this region can provide scientific evidence and practical guidance for achieving Green Space Justice and improving residents’ quality of life. This research can also serve as a valuable reference for the planning of green and blue spaces in other urban clusters across the country.

Furthermore, the built-up areas of the CZXCG are centered around the Xiang River, which allows the study to not only reveal the current state of green space distribution and utilization but also explore the moderating role of Blue Space Justice. This can provide valuable insights for further enhancing the level of Green Space Justice in urban areas.

### 2.2 Data sources

The data for this study are divided into geographic spatial data and survey questionnaire data. The geographic spatial data include administrative boundary data, population density spatial datasets, and land use remote sensing monitoring data. All of these datasets are sourced from the Data Center for Resources and Environmental Sciences, Chinese Academy of Sciences^1^. The land use remote sensing monitoring data were generated using Landsat TM/ETM and Landsat 8 imagery, with a spatial resolution of 30 m × 30 m.

The questionnaire data were collected by the research team through in-depth field visits and online surveys conducted from June 2022 to December 2022 in various communities within the built-up areas of the Chang-Zhu-Tan urban agglomeration in Hunan Province. The research sample was obtained using random sampling methods. Questionnaires were randomly distributed in communities near various typical public green parks and the Xiang River waterfront. Additionally, some online questionnaires were distributed through an online survey platform to supplement the field survey data. A total of 1,061 questionnaires were distributed, with 801 valid responses received. Among the survey respondents, 29.09% were male, and 70.91% were female. The age distribution was primarily between 31 and 40 years old, accounting for 49.19%, followed by 41–50 years old at 22.97%, reflecting high participation from middle-aged and young adults. This concentration indicates the significant demand for and utilization of green spaces by this age group in urban areas. In terms of education level, 55.93% of respondents had an associate degree or higher. Monthly income was mainly in the ranges of 0–2000 RMB and 5,000–8,000 RMB, accounting for 22.72 and 21.47%, respectively. These data comprehensively reflect the needs and usage of green spaces by residents with different backgrounds and characteristics (Table 1), providing a solid data foundation for studying the impact of Green Space Justice on public mental health.

**Table 1:** Basic information statistics of survey respondents.

Basic property	Category	Count	%
Gender	Male	233	29.09%
	Female	568	70.91%
Age	6–12	28	3.50%
	12–20	59	7.37%
	21–30	67	8.36%
	31–40	394	49.19%
	41–50	184	22.97%
	51–60	66	8.24%
	60+	3	0.37%
Record of formal schooling	Junior high school and below	100	12.48%
	High School, Higher Vocational	226	28.21%
	College, Undergraduate	448	55.93%
	Master’s Degree	27	3.37%
Monthly income	0–2000	182	22.72%
	2000–3,000	58	7.24%
	3,000–4,000	89	11.11%
	4,000–5,000	144	17.98%
	5,000–8,000	172	21.47%
	8,000–10,000	72	8.99%
	10,000–15,000	44	5.49%
	15,000+	40	4.99%

Additionally, the survey data in this study are primarily based on self-reports from respondents. This data collection method may be subject to several potential biases, including social desirability bias and recall bias. Social desirability bias may lead respondents to provide answers that align with social expectations rather than their true behaviors or feelings. Recall bias can affect the accuracy of respondents’ recollections of past behaviors or emotions. Furthermore, respondents’ emotional states, comprehension abilities, and the design of the questionnaire itself may also influence the data. To mitigate these potential biases, this study implemented the following measures: First, the questionnaire was designed using validated scales and clear question descriptions to reduce comprehension bias. Second, during data collection, respondents were ensured anonymity and encouraged to respond in a pressure-free environment, thereby minimizing the impact of social desirability bias. Additionally, in the data analysis phase, statistical methods such as control variable analysis were employed to detect and correct potential biases, thereby enhancing the validity and reliability of the data. Moreover, this study employed a random sampling method, distributing questionnaires in communities near various typical public green parks and the Xiangjiang River, to ensure diversity and representativeness of the sample. The survey data collected in the field were supplemented with data from an online survey platform to broaden the sample coverage and improve the comprehensiveness of the data.

### 2.3 Research hypotheses

**Figure 2:**
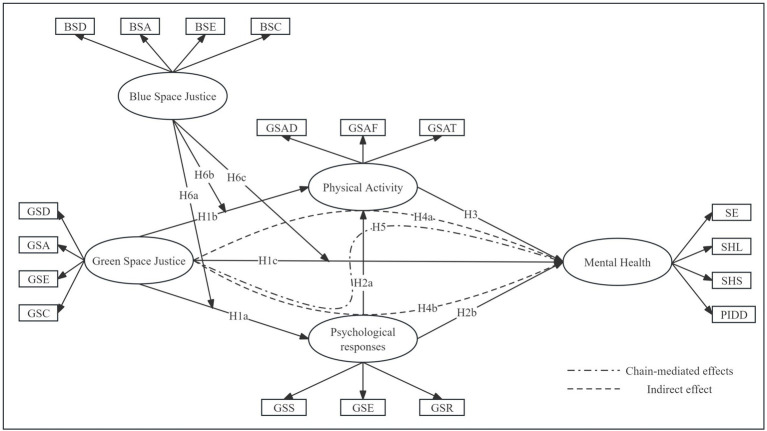
Hypothetical model construction. Abbreviations can be found in Table 2.

This study aims to explore the impact of green spaces on public physical activity, psychological responses, and mental health in urban built-up areas from a justice perspective and to examine the moderating role of Blue Space Justice (Figure 2). Based on relevant literature and theoretical frameworks, the following research hypotheses are proposed:

**Table 2:** Model variables and question items.

Variable type	Latent variable	Observed variable	Question item	Option assignment
Independent variable	Green Space Justice	Green space diversity (GSD)	Number of different green spaces accessible within the buffer	
		Green space availability (GSA)	Green space coverage area within the buffer	
		Green space equity (GSE)	Ratio of green space coverage area to community area within the buffer	
		Green space accessibility (GSC)	Distance from each community centroid to the nearest green space	
Moderating Variable	Blue Space Justice	Blue space diversity (BSD)	Number of different water bodies accessible within the buffer	
		Blue space availability (BSA)	Blue space coverage area within the buffer	
		Blue space equity (BSE)	Ratio of blue space coverage area to community area within the buffer	
		Blue space accessibility (BSC)	Distance from each community centroid to the nearest water body	
Mediating Variables	Physical Activity	Green space activity distance (GSAD)	How far is the distance from your home to the urban green space you often visit?	
		Green space activity frequency (GSAF)	How many times do you visit nearby green spaces per month?	
		Green space activity times (GSAT)	How many hours do you usually spend in nearby green spaces each visit?	
	Psychological Responses	Green space satisfaction (GSS)	How satisfied are you with the green space environment?	(Very dissatisfied = 1, Very satisfied = 5)
		Green space enjoyment (GSE)	How does the green space environment affect your emotions?	(Very unhappy = 1, Very happy = 5)
		Green space relaxation (GSR)	How does the green space environment affect your stress levels?	(Very stressed = 1, Very relaxed = 5)
Dependent Variable	Mental Health	Self-esteem (SE)	Have you recently felt a lack of self-worth?	(Strongly disagree = 1, Strongly agree = 5)
		Self-harm severity (SHS)	Have you recently had suicidal thoughts or engaged in self-harm behavior?	
		Self-happiness level (SHL)	Have you recently felt quite happy?	
		Psychological illness diagnostic degree (PIDD)	Have you recently been diagnosed with psychological disorders such as anxiety, depression, or OCD?	

Green Space Justice refers to the equitable distribution of natural space resources such as green spaces and parks within cities. When the distribution of green spaces is more balanced and fair, residents are more likely to be willing to engage in outdoor activities. This increased willingness leads to more frequent and longer participation in green space activities, which directly improves residents’ mental health. Fairly distributed green spaces provide better leisure and relaxation venues, helping to alleviate psychological stress and enhance life satisfaction and happiness. Therefore, the following hypotheses are proposed:

*H1a*: Green Space Justice has a positive impact on psychological responses.

*H1b*: Green Space Justice has a positive impact on physical activity.

*H1c*: Green Space Justice has a positive impact on mental health.

Psychological responses are closely related to physical activity and mental health. When residents’ willingness to engage in outdoor activities increases, their frequency and duration of green space activities also increase. Higher willingness to engage in outings means residents are more likely to participate in outdoor activities, thereby enhancing their psychological health. Therefore, the following hypotheses are proposed:

*H2a*: Psychological responses have a positive impact on physical activity.

*H2b*: Psychological responses have a positive impact on mental health.

Physical activity are closely related to mental health. More frequent and longer green space activities help relieve stress and improve mood, thereby enhancing residents’ mental health. Therefore, the following hypothesis is proposed:

*H3*: Physical activity have a positive impact on mental health.

Green Space Justice can influence mental health not only through its impact on residents’ psychological responses but also through its impact on their physical activity. Therefore, the following hypotheses are proposed:

*H4a*: Psychological responses mediate the relationship between Green Space Justice and mental health.

*H4b*: Physical activity mediate the relationship between Green Space Justice and mental health.

Green Space Justice influences mental health through a chain mediation effect involving psychological responses and physical activity. Therefore, the following hypothesis is proposed:

*H5*: Green Space Justice affects residents’ mental health through a chain mediation involving psychological responses and physical activity.

Blue Space Justice refers to the equitable distribution of water resources such as bodies of water and rivers within cities. Balanced distribution of blue spaces can enhance the attractiveness of green spaces, making residents more willing to engage in outdoor activities. This increases the frequency and duration of residents’ participation in green space activities and further improves the overall environmental quality of green spaces. Therefore, the following hypotheses are proposed:

*H6a*: Blue Space Justice positively moderates the relationship between Green Space Justice and psychological responses.

*H6b*: Blue Space Justice positively moderates the relationship between Green Space Justice and physical activity.

*H6c*: Blue Space Justice positively moderates the relationship between Green Space Justice and mental health.

By testing the above hypotheses, this study will reveal the importance of Green Space Justice and Blue Space Justice in enhancing public psychological responses, physical activity, and mental health. The findings will provide a theoretical basis for urban planning and public policy.

### 2.4 Variable selection

#### 2.4.1 Independent variable

**Figure 3:**
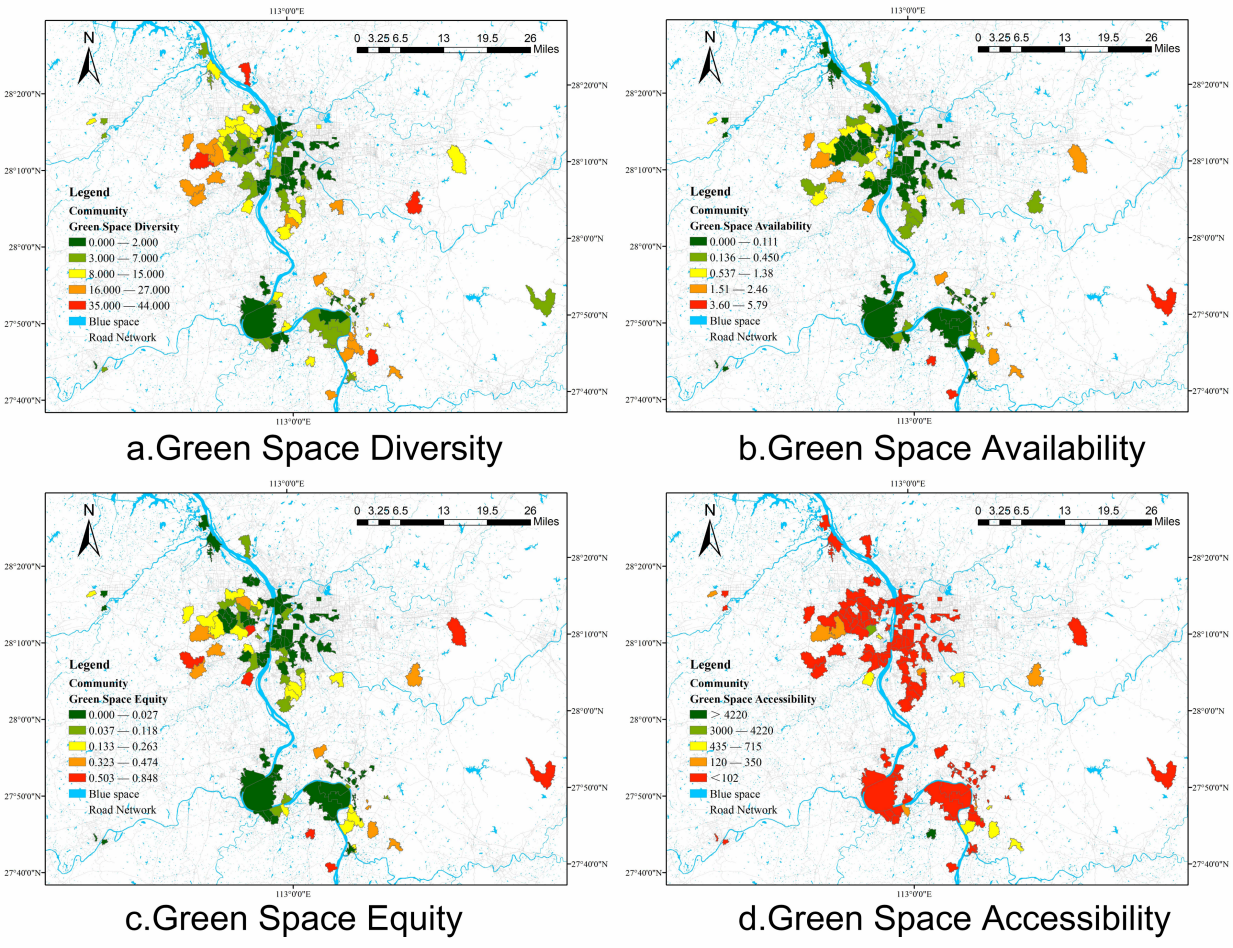
Spatial characteristics of Green Space Justice indicators in each community.

Green Space Justice is the independent variable in this study, aimed at assessing the fairness and equity of green space resource distribution within communities. Green Space Justice is composed of four measurement indicators: green space diversity, green space availability, green space equity, and green space accessibility. The comprehensive evaluation of these four indicators allows for a thorough assessment of the equity of green space resource distribution within communities, providing a solid foundation for studying the impact of Green Space Justice on public physical activity, psychological responses and mental health. This study uses buffers as spatial statistical units and communities as visualization units (Figure 3). The detailed descriptions of these four indicators are as follows:

1. Green Space Diversity: green space diversity measures the number of different green spaces accessible within a 30-min walking distance from the community centroid. According to Wolch et al. (75) and the Ministry of Housing and Urban–Rural Development of the People’s Republic of China (2019), urban green spaces include forests, grasslands, and more. Specifically, the number of different green spaces within a 1,500-meter radius buffer zone from the community centroid represents the green space diversity of that community. Diverse green spaces not only meet the varied recreational needs of residents but also enhance the overall quality of the community environment. Studies have shown that green space diversity is positively correlated with residents’ mental health and social interactions (76, 77).
2. Green Space Availability: green space availability refers to the area of green space within the buffer zone that is accessible to residents. The larger the green space area, the richer the green space resources available for residents, providing more outdoor activities and leisure space.
3. Green Space Equity: green space equity is the ratio of green space area to the total area of the buffer zone. A higher ratio indicates a more balanced distribution of green spaces within the buffer zone, ensuring fair opportunities for residents to access green space resources. Research has found that equitable distribution of green spaces plays a significant role in improving residents’ life satisfaction and social cohesion (28, 78).
4. Green Space Accessibility: green space accessibility is the distance from each community centroid to the nearest green space. To more accurately measure green space accessibility, this study employs the enhanced two-step floating catchment area (2SFCA) method. Walking is the most common mode of daily transportation, and a 30-min walk can be considered the limit of walking accessibility. At a normal walking speed, a person can walk approximately 2,500 meters in 30 min (79). Therefore, this study selects a buffer zone with a radius of 1,500 m, capturing all green spaces that can be reached during daily walking. Green spaces within this range are also suitable for visits using public transportation or cycling. Research shows that high accessibility to green spaces can significantly improve residents’ mental and physical health (80, 81).

#### 2.4.2 Mediating variables

This study considers physical activity and psychological responses as mediating variables in the impact of Green Space Justice on mental health. These variables are composed of several specific indicators:

1. Physical Activity measures residents’ actual participation in green space activities, including green space activity distance, frequency, and duration. The specific explanations are as follows: Green space activity distance reflects the ease of access to green spaces for residents; shorter distances indicate easier access; Green space activity frequency reflects how often residents engage in green space activities; higher frequency indicates more frequent use of green space resources; Green space activity duration reflects the amount of time residents spend in green spaces; longer durations indicate more time spent in green environments.
2. Psychological Responses measure residents’ subjective perceptions of green spaces, including satisfaction, enjoyment, and relaxation with green spaces. These indicators are collected through questionnaires, using a five-point Likert scale to quantify residents’ psychological response levels. The specific explanations are as follows: Green space satisfaction reflects residents’ overall evaluation of the green environment; higher satisfaction indicates greater contentment with green spaces; Green space enjoyment measures the emotional impact of green spaces on residents; higher enjoyment indicates more positive emotions experienced in green spaces; Green space relaxation measures the stress-relief effect of green spaces on residents; higher relaxation indicates more stress and pressure relief experienced in green spaces.

#### 2.4.3 Moderating variable

**Figure 4:**
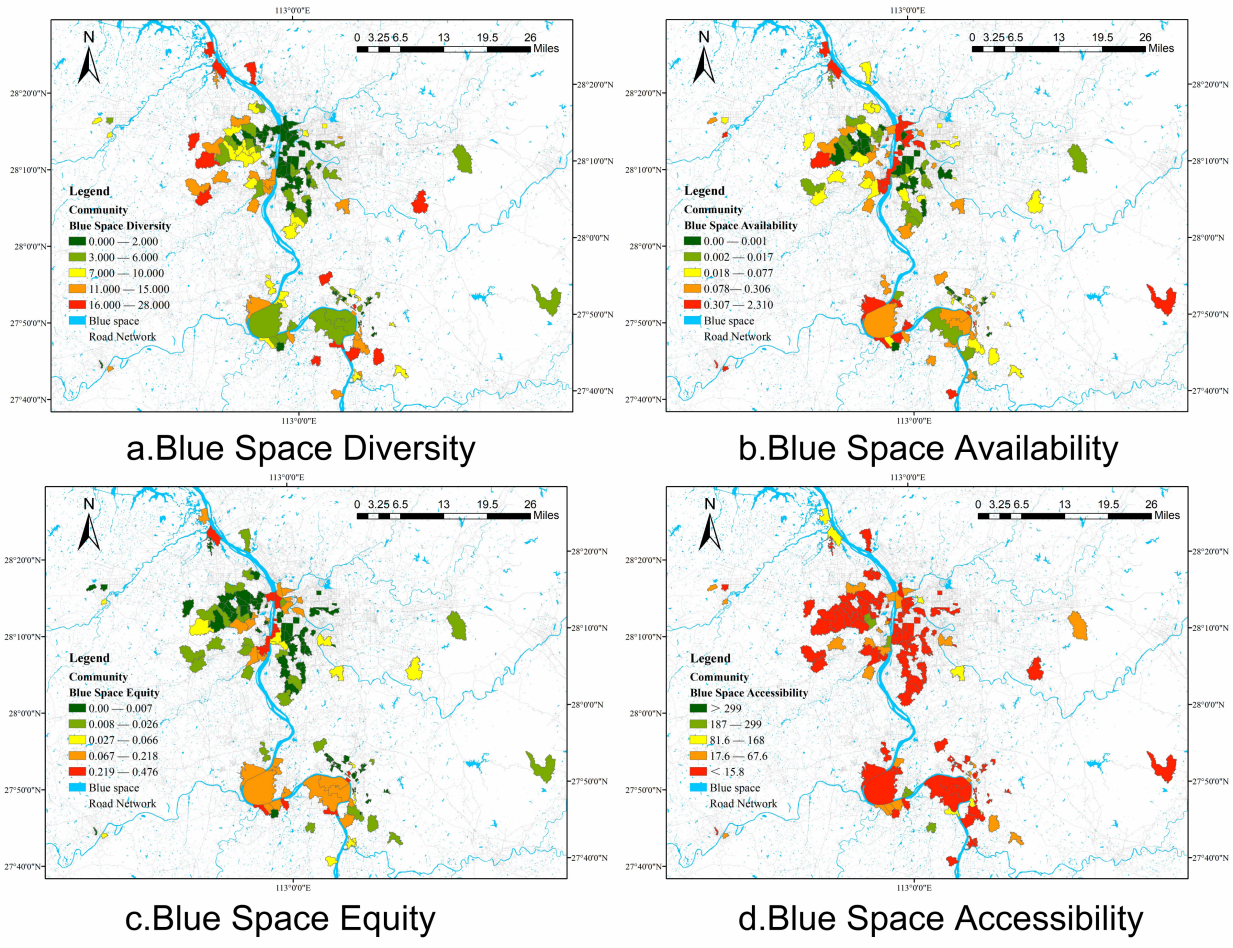
Characteristics of Blue Space Justice indicators in each community.

Blue Space Justice serves as the moderating variable in this study, aimed at assessing the fairness and equity of blue space resource distribution within communities. Blue Space Justice is composed of four measurement indicators: blue space diversity, blue space availability, blue space equity, and blue space accessibility. The measurement methods for these indicators are the same as those for Green Space Justice (Figure 4).

#### 2.4.4 Dependent variable

Mental health is the dependent variable in this study, measured by four indicators: self-worth, happiness, self-harm, and psychological illness diagnosis. Self-worth reflects an individual’s subjective evaluation of their value, including their sense of self-esteem and confidence (82). Happiness reflects an individual’s overall subjective evaluation of their life satisfaction and happiness, serving as an important indicator of mental health (83, 84). Self-harm reflects whether an individual has thoughts of suicide or self-harm behavior, assessing self-injurious actions (85). Psychological illness diagnosis reflects whether an individual has been diagnosed with psychological illnesses, including anxiety, depression, obsessive-compulsive disorder, and other psychological conditions (86). These indicators are collected through questionnaires using a five-point Likert scale (1 = strongly disagree, 5 = strongly agree) to quantify the individual’s mental health level (Table 2).

### 2.5 Research methods

#### 2.5.1 Improved Gaussian-based two-step floating catchment area (2SFCA) method

The improved Gaussian-based 2SFCA method is a commonly used spatial analysis method for assessing the impact of urban green spaces on residents’ mental health. This method first calculates the distance from the community centroid to the nearest blue or green space, applying the improved 2SFCA method for distance weighting to consider green space accessibility. Secondly, starting from the community centroid, all green spaces within a 1,500-meter radius buffer zone are regarded as accessible green spaces to evaluate the frequency and duration of green space activities. The advantage of this method lies in its ability to account for the accessibility and convenience of green space activities, providing a more accurate assessment of their impact on mental health (87, 88). The specific calculation process is as follows:

Step 1: Calculate the Supply Capacity of Each Service Facility (Equations 1, 2).


(1) $$S_{j}=\frac{1}{\sum_{k\in D_{j}}f\left(d_{jk}\right)}$$

Where *S*_j_ is the supply capacity of service facility *j*, *D*_j_ is the service area of facility *j*, *f*(*d*_jk_) is the distance decay function (typically a Gaussian function):


(2) $$f\left(d_{jk}\right)=e^{-\frac{d_{jk}^{2}}{2\sigma^{2}}}$$

Here, *d*_jk_ is the distance from service facility *j* to demand point *k*, and σ is the distance decay parameter.

Step 2: Calculate the Accessibility Index of Each Demand Point (Equation 3).


(3) $$A_{i}=\underset{j\in S_{i}}{\sum }S_{j}.f\left(d_{ij}\right)$$

Where *A*_i_ is the accessibility index of demand point *i*, *S*_i_ is the service area accessible to demand point *i*, and *f*(*d*_ij_) is the distance decay function indicating the distance decay from demand point *i* to service facility *j*.

#### 2.5.2 Structural equation modeling (SEM)

Structural equation modeling (SEM) includes measurement models and structural models, representing the relationships between observed variables and latent variables, and the relationships among latent variables, respectively. SEM is used to assess the relationships among Green Space Justice, physical activity, psychological responses, and mental health. This model integrates the direct and indirect relationships among variables and uses path analysis and model fitting to validate the research hypotheses. In this study, SEM is employed to explore the direct and indirect effects of Green Space Justice on mental health, and further analyze the mediating mechanisms and moderating effects (89, 90).

1. Measurement model (Equations 4, 5):


(4) $$X=\wedge_{X}\xi +\delta$$

(5) $$Y=\wedge_{Y}\eta +\in$$

Where *X* and *Y* are vectors of observed indicators, ξ and η are vectors of latent variables, $\wedge_{X}$ and $\wedge_{Y}$ are factor loading matrices, and δ and ϵ are error terms.

2. Structural model (Equation 6):


(6) η=Bη+Γξ+ς

Where *η* is the vector of endogenous latent variables, ξ is the vector of exogenous latent variables, B is the matrix of path coefficients among endogenous latent variables, Г is the matrix of path coefficients from exogenous to endogenous latent variables, and ς is the error term of the structural model.

#### 2.5.3 Moderation effect analysis

Moderation effect analysis is a statistical method used to explore mediation and moderation effects, often revealing complex relationships in research. In this study, moderation effect analysis is employed to examine whether the impact of Green Space Justice on physical activity, psychological responses, and mental health is moderated by the characteristics of Blue Space Justice.

## 3 Results

### 3.1 Reliability and validity testing of the scales

This study used reliability analysis and factor analysis to test the reliability and validity of the questionnaire data. The Cronbach’s *α* values for psychological responses and mental health were 0.666 and 0.534, respectively. Since both α values are greater than 0.5, the reliability is considered good, indicating a high level of internal consistency within the variables. The KMO test values for psychological responses and mental health were 0.531 and 0.647, respectively. With KMO test statistics above 0.5, the model is suitable for factor analysis, proving that the questionnaire has good validity.

### 3.2 Structural equation modeling fit testing

In terms of model fit evaluation, the higher the model fit, the more meaningful the parameter estimates. A comprehensive evaluation of the model fit was conducted using all indicators. The overall SEM model fit test results are shown in Table 3. According to the analysis results, the scores for GFI, AGFI, NFI, IFI, CFI, and TLI were all higher than 0.8, and the scores for RMSEA and RMR were both lower than 0.08, indicating that the scores are within a reasonable range. Therefore, the model’s fit is good, and path regression analysis can be conducted.

**Table 3:** Model fit results.

Fit index	Description	Reasonable range	Result	Evaluation
GFI	Goodness-of-Fit Index	>0.8, reasonable; >0.9, good	0.977	Good
AGFI	Adjusted Goodness-of-Fit Index	>0.8, reasonable; >0.9, good	0.966	Good
RMR	Root Mean Square Residual	<0.08, reasonable; <0.05, good	0.022	Good
RMSEA	Root Mean Square Error of Approximation	<0.08, reasonable; <0.05, good	0.029	Good
NFI	Normed Fit Index	>0.8, reasonable; >0.9, good	0.976	Good
IFI	Incremental Fit Index	>0.8, reasonable; >0.9, good	0.991	Good
CFI	Comparative Fit Index	>0.8, reasonable; >0.9, good	0.991	Good

### 3.3 Descriptive statistical analysis

Since the data for the independent and moderating variables have already been presented in Section 2. Materials and Methods, this subsection focuses on the descriptive statistical results of the questionnaire indicators. As shown in Table 4, the results are as follows:

**Table 4:** Descriptive statistics of the questionnaire indicators.

	Mean	Std	Min	25%	50%	75%	Max
GSAF	4.398	6.091	1	1	2	4	30
GSAT	2.122	1.305	0.1	1	2	3	10
GSAD	2.030	1.542	0.02	1	1.5	3	6
GSE	4.266	0.652	1	4	4	5	5
GSR	4.263	0.636	2	4	4	5	5
GSS	4.271	0.665	1	4	4	5	5
SE	2.429	1.027	1	2	2	3	5
SHL	3.542	0.888	1	3	3	4	5
SHS	1.577	0.965	1	1	1	2	5
PIDD	1.619	0.980	1	1	1	2	5

First, GSAD represents the distance from home to the frequently visited urban green space, with a mean value of 2.030 km and a standard deviation of 1.542 km, indicating that most individuals live relatively close to the green spaces they visit. GSAF represents the number of visits to nearby green spaces per month, with a mean value of 4.398 and a standard deviation of 6.091, showing a wide variation in the frequency of green space visits among the sample. GSAT represents the duration of each visit to nearby green spaces, with a mean value of 2.122 h and a standard deviation of 1.305 h, indicating that the time most individuals spend in green spaces is fairly consistent.

Second, GSS measures individuals’ satisfaction with the green space environment, with a mean value of 4.271 and a standard deviation of 0.665, suggesting that most individuals are highly satisfied with the green space environment. GSE measures the impact of the green space environment on emotions, with a mean value of 4.266 and a standard deviation of 0.652, indicating that most individuals experience positive emotional uplift in green spaces. GSR measures the extent to which green spaces relieve stress, with a mean value of 4.263 and a standard deviation of 0.636, showing that the majority of respondents experience significant relaxation in green spaces.

Lastly, SE measures whether individuals have recently felt a lack of self-worth, with a mean value of 2.429 and a standard deviation of 1.027, indicating some variation in self-esteem levels within the sample. SHS reflects whether individuals have recently had suicidal thoughts or engaged in self-harm behaviors, with a mean value of 1.577 and a standard deviation of 0.965, showing that while the overall severity of self-harm is low, there are some individual differences. SHL measures individuals’ recent happiness levels, with a mean value of 3.542 and a standard deviation of 0.888, indicating that happiness levels are fairly concentrated among the sample. PIDD measures whether individuals have recently been diagnosed with psychological disorders such as anxiety, depression, or obsessive-compulsive disorder (OCD), with a mean value of 1.619 and a standard deviation of 0.980, suggesting that a portion of the sample does have mental health issues.

### 3.4 Model estimation results analysis

Table 5 reports the parameter estimation results of the measurement model. The standardized factor loadings between latent variables and observed variables are positive, ranging from 0.164 to 0.483. The parameter estimates of the observed variables for each latent variable are significant at the 1% level, indicating good explanatory power of the observed variables for the latent variables.

**Table 5:** Path coefficients.

Path	Unstandardized coefficient	Standardized coefficient	S.E.	C.R.	*P*
Green Space Justice → Psychological Response	0.534	0.483	0.054	9.808	***
Green Space Justice → Physical Activity	0.42	0.387	0.058	7.195	***
Psychological Response → Physical Activity	0.161	0.164	0.051	3.124	**
Physical Activity → Mental Health	0.327	0.328	0.044	7.42	***
Psychological Response → Mental Health	0.26	0.266	0.044	5.969	***
Green Space Justice → Mental Health	0.314	0.29	0.051	6.194	***

*** indicates significance at the 0.001 level; ** indicates significance at the 0.01 level.

**Figure 5:**
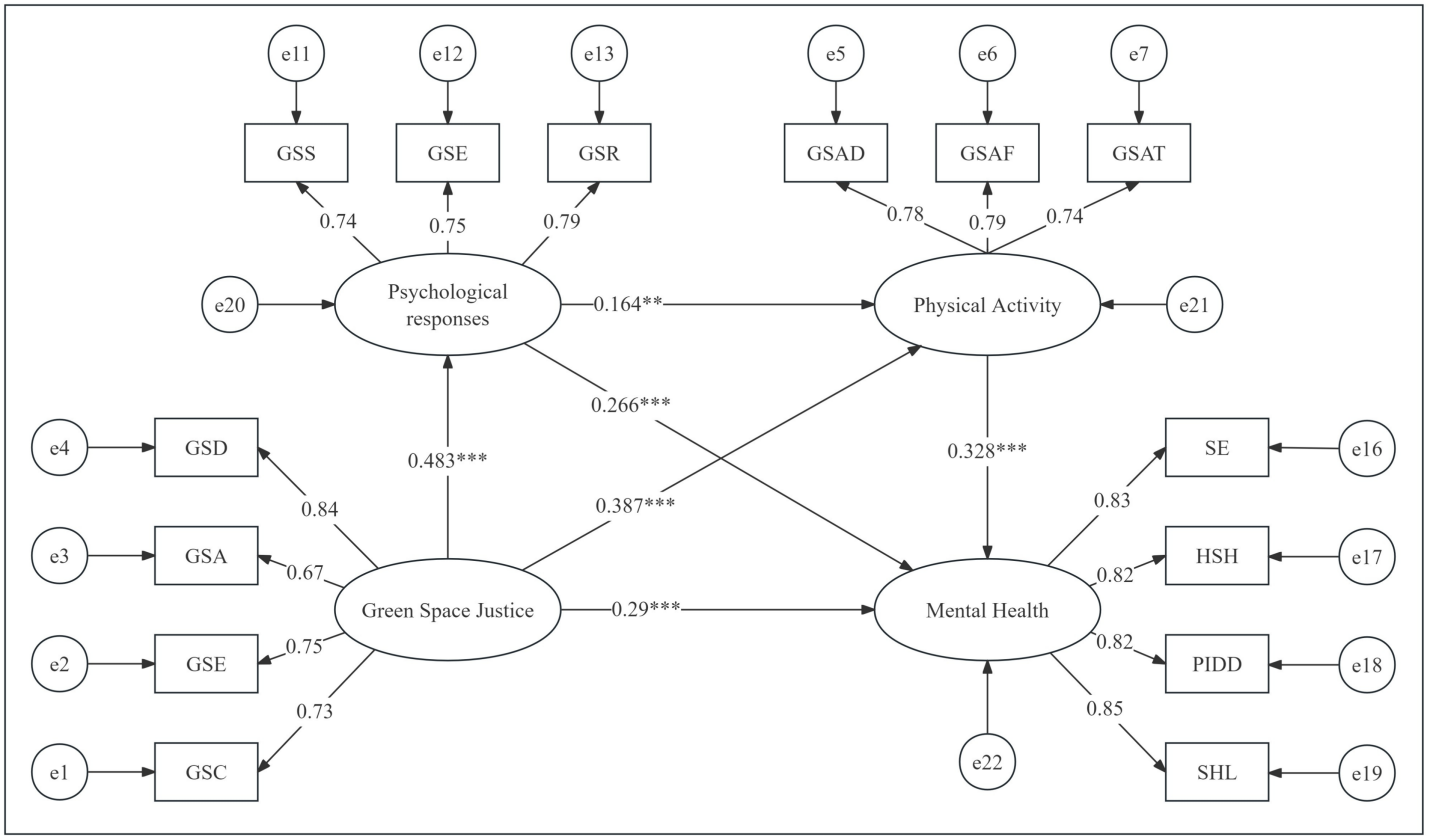
The path coefficients of structural equation modeling.

Figure 5 presents the parameter estimation results of the structural model. It shows that the three variables—Green Space Justice, psychological responses, and physical activity—each have a significant impact on the mental health of urban residents.

At the 1% significance level, a 1-unit increase in Green Space Justice leads to a 0.483-unit increase in psychological responses. This indicates that higher justice in the distribution of green spaces results in more positive psychological responses from residents, manifested as increased satisfaction, joy, and relaxation with green spaces. This result supports H1a.

Green Space Justice also has a significant positive impact on physical activity (*β* = 0.387, *p* < 0.001). This means that higher Green Space Justice increases the frequency and duration of residents’ participation in green space activities. This result supports H1b, indicating that Green Space Justice can significantly increase residents’ physical activity.

Psychological responses have a significant positive impact on physical activity (*β* = 0.164, *p* = 0.002). This shows that positive psychological responses to green spaces can promote more participation in green space activities. This result supports H2a, indicating that psychological responses partially mediate the relationship between Green Space Justice and physical activity.

Physical activity has a significant positive impact on mental health (*β* = 0.328, *p* < 0.001). This indicates that more participation in green space activities can significantly improve residents’ mental health, including enhancing self-worth and happiness, and reducing self-harm behaviors and the diagnosis rate of psychological illnesses. This result supports H3, showing that physical activity has an important positive impact on mental health.

Psychological responses have a significant positive impact on mental health (*β* = 0.266, *p* < 0.001). This means that positive psychological responses to green spaces can directly improve residents’ mental health. This result supports H2b.

Green Space Justice has a significant positive impact on mental health (*β* = 0.29, *p* < 0.001). This indicates that Green Space Justice can directly enhance residents’ mental health. This result supports H1c, showing that Green Space Justice not only affects mental health through psychological responses and physical activity but also has a direct positive impact.

### 3.5 Mediation effect analysis

This study used the SEM model to test the mediation effects. The results, shown in Table 6, demonstrate the mediation effects of Green Space Justice on mental health through psychological responses and physical activity, along with their respective proportions.

**Table 6:** Mediation effect test results.

Variable	Effect value	Lower	Upper	*P*
Mediation 1	0.139	0.098	0.191	<0.01
Mediation 2	0.137	0.1	0.191	<0.01
Chain Mediation 3	0.028	0.013	0.044	<0.01
Direct Effect	0.314	0.226	0.392	<0.01
Total Effect	0.619	0.533	0.704	<0.01
Mediation Proportion 1	0.225	0.163	0.308	<0.01
Mediation Proportion 2	0.222	0.158	0.311	<0.01
Chain Mediation Proportion 3	0.045	0.02	0.074	<0.01

Mediation Path 1: Green Space Justice → Physical Activity → Mental Health; Mediation Path 2: Green Space Justice → Psychological Responses → Mental Health; Chain Mediation Path 3: Green Space Justice → Psychological Responses → Physical Activity → Mental Health.

According to Table 6, the total effect and direct effect of Green Space Justice on mental health are significant, with effect values of 0.619 and 0.314, respectively (*p* < 0.01). This indicates that Green Space Justice has a significant positive impact on residents’ mental health through both direct and indirect effects. Even without considering the mediating variables, Green Space Justice can directly enhance residents’ mental health.

Specifically, the mediation effect of Green Space Justice on mental health through physical activity is significant, with an effect value of 0.139 (*p* < 0.01), accounting for 22.5% of the total effect. This suggests that Green Space Justice can improve mental health by increasing the frequency and duration of residents’ green space activities. This result supports H4b. The mediation effect of Green Space Justice on mental health through psychological responses is also significant, with an effect value of 0.137 (*p* < 0.01), accounting for 22.2% of the total effect. This indicates that Green Space Justice can improve mental health by enhancing residents’ psychological responses to green spaces. This result supports H4a. Additionally, the chain mediation effect of Green Space Justice on mental health through psychological responses and physical activity is significant, with an effect value of 0.028 (*p* < 0.01), accounting for 4.5% of the total effect. This shows that Green Space Justice can first enhance residents’ psychological responses and then increase green space activities, ultimately improving mental health. This result supports H5.

Overall, Green Space Justice has a significant positive impact on mental health through both direct and mediation effects. The mediation effects through psychological responses and physical activity are substantial, indicating that these two variables play important roles in the impact of Green Space Justice on mental health. Although the chain mediation effect is relatively small, it is still significant, demonstrating that the combined pathway of psychological responses and physical activity is also an important mechanism through which Green Space Justice affects mental health.

### 3.6 Moderation effect analysis

**Figure 6:**
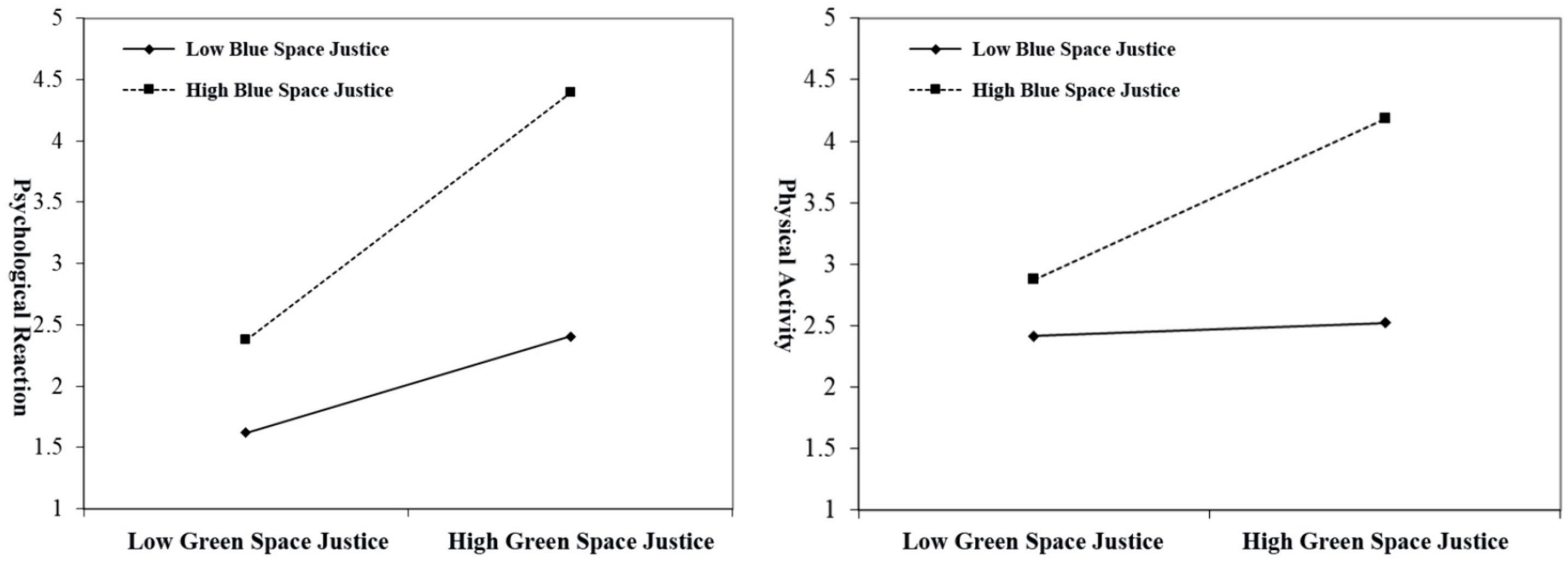
Effects of Green Space Justice on psychological responses and physical activity at different levels of Blue Space Justice.

This study used the Process method in SPSS to test the moderation effects of Green Space Justice and Blue Space Justice. The results are shown in Figure 6 and Table 7.

**Table 7:** Moderation effect analysis.

Variable	Mental Health (Y)	Physical Activity (M1)	Psychological Response (M2)	Mental Health (Y)
(Intercept)	3.409*** −0.023	3.271*** −0.015	3.260*** −0.016	3.409*** −0.018
Green Space Justice (X)	0.766*** −0.03	0.744*** −0.02	0.827*** −0.02	0.229*** −0.045
Blue Space Justice (W)		0.160*** −0.019	0.161*** −0.02	0.164*** −0.025
Green Space Justice (X): Blue Space Justice (W)		0.336*** −0.025	0.341*** −0.026	0.011 −0.035
Physical Activity (M1)				0.319*** −0.068
Psychological Response (M2)				0.364*** −0.066
*R*^2^	0.491	0.707	0.734	0.679
Adj. R2	0.490	0.706	0.733	0.677

Unstandardized regression coefficients are displayed, with standard errors in parentheses. *p < 0.05, **p < 0.01, ***p < 0.001.

Table 7 presents the moderation effect analysis results of Green Space Justice (X), Blue Space Justice (W), psychological responses (M2), and physical activity (M1) on mental health (Y). Specifically: Green Space Justice has a significant positive impact on psychological responses (*β* = 0.827, *p* < 0.001). Blue Space Justice also has a significant positive impact on psychological responses (*β* = 0.161, *p* < 0.001). The interaction term between Green Space Justice and Blue Space Justice has a significant positive impact on psychological responses (*β* = 0.341, *p* < 0.001), indicating that Blue Space Justice can enhance the positive effect of Green Space Justice on psychological responses. This result supports H6a. Green Space Justice has a significant positive impact on physical activity (*β* = 0.744, *p* < 0.001). Blue Space Justice also has a significant positive impact on physical activity (*β* = 0.160, *p* < 0.001). The interaction term between Green Space Justice and Blue Space Justice has a significant positive impact on physical activity (*β* = 0.336, *p* < 0.001), indicating that Blue Space Justice can enhance the positive effect of Green Space Justice on physical activity. This result supports H6b. Green Space Justice has a significant positive impact on mental health (*β* = 0.229, *p* < 0.001). Physical activity has a significant positive impact on mental health (*β* = 0.319, *p* < 0.001). Psychological responses have a significant positive impact on mental health (*β* = 0.364, *p* < 0.001). However, the interaction term between Green Space Justice and Blue Space Justice does not have a significant impact on mental health (*β* = 0.011, *p* = 0.710), indicating that Blue Space Justice does not significantly moderate the effect of Green Space Justice on mental health. This result does not support H6c.

Additionally, Figure 6 shows the effects of Green Space Justice on psychological responses and physical activity at different levels of Blue Space Justice. It can be seen that when Blue Space Justice is high, the positive effects of Green Space Justice on psychological responses and physical activity are more pronounced, further supporting the results of the moderation effect analysis.

Overall, this study finds that Blue Space Justice can significantly moderate the effects of Green Space Justice on psychological responses and physical activity, but not on mental health (Table 8).

**Table 8:** Hypothesis testing results.

Hypotheses		Result
H1a	Green Space Justice positively affects psychological responses	Supported
H1b	Green Space Justice positively affects physical activity	Supported
H1c	Green Space Justice positively affects mental health	Supported
H2a	Psychological responses positively affect physical activity	Supported
H2b	Psychological responses positively affect mental health	Supported
H3	Physical activity positively affects mental health	Supported
H4a	Psychological responses mediate the relationship between Green Space Justice and mental health	Supported
H4b	Physical activity mediates the relationship between Green Space Justice and mental health	Supported
H5	Green Space Justice affects mental health through a chain mediation involving psychological responses and physical activity	Supported
H6a	Blue Space Justice positively moderates the relationship between Green Space Justice and psychological responses	Supported
H6b	Blue Space Justice positively moderates the relationship between Green Space Justice and physical activity	Supported
H6c	Blue Space Justice positively moderates the relationship between Green Space Justice and mental health	Not Supported

## 4 Discussion

### 4.1 Key findings

This study employed the improved Gaussian-based two-step floating catchment area (2SFCA) method, structural equation modeling (SEM), and moderation effect analysis to explore the impact of Green Space Justice on public physical activity and mental health, and to examine the moderating role of Blue Space Justice. The results show that Green Space Justice has a significant positive impact on psychological responses, physical activity, and mental health; psychological responses and physical activity mediate the relationship between Green Space Justice and mental health; Blue Space Justice significantly moderates the effects of Green Space Justice on psychological responses and physical activity but does not significantly moderate its direct impact on mental health.

### 4.2 Direct effects of Green Space Justice

The findings indicate that Green Space Justice has a significant positive impact on psychological responses, physical activity, and mental health. This aligns with existing research, suggesting that the fair distribution of green space resources can improve residents’ life satisfaction and happiness (91–93). Other studies have shown that residents living near green spaces have higher levels of physical activity and better physical health (94), with these effects being evident across all age groups, especially among middle-aged and older adults (95). Specifically, Green Space Justice increases the diversity and availability of green spaces within communities, making it easier for residents to access natural environments, thereby enhancing their psychological satisfaction and relaxation, increasing the frequency and duration of their green space activities, and ultimately improving their overall mental health.

### 4.3 Mediation and chain mediation effects of psychological responses and physical activity

The study further finds that psychological responses and physical activity play important mediating roles between Green Space Justice and mental health. Firstly, Green Space Justice enhances residents’ psychological responses, increasing their willingness to engage in green space activities, which in turn increases actual physical activity levels. This finding is consistent with previous research, indicating that psychological satisfaction and relaxation are key drivers of green space utilization (76, 95). Secondly, frequent and prolonged green space activities significantly improve residents’ mental health (94), including enhancing self-worth and happiness, reducing self-harm behaviors, and decreasing the incidence of psychological illnesses (96). Numerous studies support these findings, such as van den Berg et al. (97), which demonstrated a significant association between frequent green space visits and lower levels of depression and anxiety, indicating that green spaces can help prevent mental illnesses to some extent.

Additionally, chain mediation effect analysis shows that Green Space Justice first enhances residents’ psychological responses, which then increases the frequency and duration of green space activities, ultimately significantly improving mental health. This emphasizes the important mediating roles of psychological responses and physical activity in the relationship between Green Space Justice and mental health. This finding is also consistent with existing research. Dadvand et al. (98) found that the relationship between green spaces and health is partly mediated by psychological health status, social support, and physical activity. Zhang et al. (99) conducted a systematic review of the pathways between green space exposure and mental health, identifying psychological restoration, air quality, and physical activity as key mediating mechanisms. Other studies have shown that the psychological restorative functions and social cohesion provided by green spaces are important pathways linking green space exposure and mental health across different cultural and environmental contexts (77, 100). This finding further supports the application of complex pathways in environmental psychology, providing new perspectives for understanding the impact of natural environments on health.

### 4.4 Moderating role of Blue Space Justice

Blue Space Justice significantly moderates the effects of Green Space Justice on psychological responses and physical activity, indicating that the positive effects of Green Space Justice are more pronounced when Blue Space Justice is higher. This is similar to findings in other studies. For instance, Pasanen et al. (101) found that living near coastal or freshwater areas is associated with better mental health, with outdoor physical activity partially mediating this relationship. Vert et al. (102) discovered that short walks in blue spaces significantly improve mental health and emotional states, highlighting the psychological restorative effects of blue spaces. Yen et al. (103) conducted a systematic review and meta-analysis, showing that physical activity in blue and green spaces has significant positive effects on mental and physical health, and the presence of blue spaces can amplify the positive effects of green spaces on health. These studies suggest that the presence and equitable distribution of blue spaces (e.g., rivers and lakes) can increase the attractiveness and utilization of green spaces. However, this study finds that Blue Space Justice does not significantly moderate the direct impact of Green Space Justice on mental health. This result warrants further exploration and analysis. First, within the study area, green spaces may play a more significant role in residents’ daily lives. Compared to green spaces, the distribution of blue spaces might be more limited, with residents having less frequent interaction with and reliance on blue spaces. This could explain why the impact of Blue Space Justice on moderating mental health is less pronounced than that of Green Space Justice (95). The natural resources and urban planning within the study area may prioritize the development of green spaces, thereby reducing the relative importance of blue spaces in daily life. Second, there may be certain limitations in the measurement of Blue Space Justice. Although this study measured Blue Space Justice through the number of accessible water bodies and their coverage area within the buffer zones, this approach may not fully capture the subtle effects of blue spaces on residents’ mental health. The influence of blue spaces may depend not only on their quantity and area but also on factors such as water quality, aesthetic appeal, and the nature of residents’ interactions with the water bodies. As a result, the current measurement approach may not fully reflect the actual role of Blue Space Justice (104). Additionally, the utilization and perception of green and blue spaces may vary significantly across different cultural and environmental contexts. In the study area, residents might be more inclined to use green spaces for leisure and physical activities, while their engagement with and perception of blue spaces might be relatively weaker (101). This cultural and environmental specificity could diminish the role of Blue Space Justice in moderating the relationship between Green Space Justice and mental health. Future research should further investigate these potential reasons or consider validating these findings in different urban contexts and cultural environments to gain a more comprehensive understanding of the combined effects of blue and green spaces on residents’ mental health.

### 4.5 Implications and significance

This study highlights the important roles of Green Space Justice and Blue Space Justice in enhancing residents’ mental and physical health, providing scientific evidence for urban planning and public policy. Specific recommendations include: (1) Improve the equity and accessibility of green spaces: urban planners should prioritize the balanced distribution of green spaces, ensuring that all community residents have convenient access to high-quality green spaces. (2) Integrating Blue Space Resources: Urban planning should fully consider the distribution of blue spaces, enhancing their synergy with green spaces to maximize the positive impact of the natural environment on residents’ health. 3.Promoting Residents’ Psychological and Physical Activities: By increasing the diversity and availability of green and blue spaces, urban planners can enhance residents’ psychological satisfaction and the frequency and duration of their green space activities, thereby improving their overall health.

To better achieve green and Blue Space Justice, the study offers the following recommendations to the government and relevant planning departments: (1) Utilize GIS technology and big data analysis: evaluate the distribution of urban green and blue spaces and the needs of residents, optimize spatial layouts, and ensure service coverage for all communities. These technological tools can help identify and address service gaps, ensuring the equitable distribution of green and blue spaces. (2) Enhance the facilities and services of existing spaces: Improve the quality of services in green and blue spaces by increasing greenery, improving walkways, and adding facilities such as children’s play areas and older adult recreation zones, to meet the physical activity needs of residents of different ages and requirements, thereby promoting their physical and mental health. 3.Encourage community involvement in space planning and management: Engage residents through public consultation and feedback, ensuring that the design and operation of green and blue spaces meet the actual needs of the community. However, effective mechanisms must be established to manage resident participation, ensuring that their suggestions and feedback are constructive and practically implementable.

In implementing the above recommendations, the following challenges may arise: (1) Complexity of resource allocation: the distribution of green and blue spaces may be influenced by environmental factors, socio-economic factors, and historical development patterns, resulting in some areas having richer park resources than others. For example, in Beijing, China, the equity of urban green spaces exhibits a clear concentric pattern, with the northern regions having better access than the southern ones, and the western regions outperforming the eastern ones. Similar imbalances may exist in other cities. (2) Accessibility constraints due to transportation networks: The accessibility of green and blue spaces may be affected by factors such as the coverage of public transportation, road conditions, and traffic congestion. As urban development progresses to later stages, making large-scale adjustments to the transportation network may be challenging, potentially impacting the accessibility and equity of green and blue spaces. (3) Limitations of public participation: while encouraging community involvement in the planning and management of green and blue spaces is a positive initiative, residents may lack the professional knowledge required to make accurate recommendations. Therefore, the government and relevant departments need to design and implement effective public participation mechanisms and communication channels to ensure that public participation is both effective and feasible.

### 4.6 Limitations and future directions

Although this study provides important findings, there are some limitations and potential weaknesses. First, the study’s data are primarily drawn from the Chang-Zhu-Tan urban agglomeration, which may limit the generalizability of the results, as they may not be broadly representative. Future research could expand to other urban regions to verify the generalizability of these findings. Second, the use of cross-sectional data in this study makes it difficult to establish causal relationships. Longitudinal studies in the future could further validate these causal relationships.

Additionally, the self-reported data used in the study may be subject to biases, such as social desirability bias and recall bias, which could affect the accuracy of the data. The limitations of the questionnaire design and potential sampling biases (e.g., the choice of communities, which may limit the generalizability of the results) also need to be further addressed and controlled in future research.

This study primarily focuses on the impact of green and blue spaces on residents’ health but does not fully account for other potential confounding variables. For instance, factors such as air quality, noise levels, social support, and community safety may also significantly influence mental health and physical activity, yet these variables were not measured in this study. Moreover, socio-economic factors (105), lifestyle (106), genetic and biological factors (107), and the surrounding environment (108, 109) may be stronger predictors of mental and physical health. Future research should incorporate these variables to build a more comprehensive analytical framework.

Finally, although this study demonstrates significant positive effects of green and blue spaces on mental health and physical activity, these results may also be limited by cultural and environmental specificity. Residents from different cultural and social backgrounds may perceive and utilize green and blue spaces differently. Therefore, cross-cultural research in the future would help verify the generalizability and stability of these findings.

## 5 Conclusion

This study systematically explored the impact of Green Space Justice on public physical activity and mental health and examined the moderating role of Blue Space Justice. The results show that Green Space Justice has a significant positive impact on psychological responses, physical activity, and mental health. Psychological responses and physical activity play important mediating roles between Green Space Justice and mental health, with Green Space Justice significantly affecting mental health through a chain mediation pathway involving psychological responses and physical activity. Additionally, Blue Space Justice significantly moderates the effects of Green Space Justice on psychological responses and physical activity, but does not significantly moderate its direct impact on mental health.

Theoretically, this study refines the concept of Green Space Justice, revealing its mechanisms in influencing mental health through complex pathways and expanding the research dimensions of Blue Space Justice. Practically, the results suggest that urban planning should focus on the equitable distribution and high accessibility of green and blue spaces to enhance residents’ psychological and physical health.

However, this study also has some limitations. First, the research data were primarily collected from the Chang-Zhu-Tan urban agglomeration, which may limit the generalizability of the findings. Second, the study employs a cross-sectional design, making it difficult to establish causal relationships. Future research should consider using longitudinal designs to better validate these causal relationships. Additionally, this study mainly focuses on the environmental factors of green and blue spaces and does not fully account for other potential socio-economic factors, such as income level and educational background, which could significantly impact mental and physical health.

Future research directions should include the following aspects: First, expanding the scope of the study to cover different cultural backgrounds and geographic regions to validate the generalizability of the findings. Second, employing longitudinal study designs to better elucidate causal relationships. Finally, future research should incorporate more socio-economic factors, such as social support and community safety, to build a more comprehensive analytical framework and to explore the multidimensional impacts of green and blue spaces on residents’ health.

## Data Availability

The raw data supporting the conclusions of this article will be made available by the authors, without undue reservation.

## Footnotes

^1^ http://www.resdc.cn
